# Supplementary figures and images for: Composition, taxonomy and functional diversity of the oropharynx microbiome in individuals with schizophrenia and controls
Source: PeerJ. 2015 Aug 25;3:e1140. doi: 10.7717/peerj.1140 (PMC4556144; doi:10.7717/peerj.1140)

**A****Alpha Diversity Measure**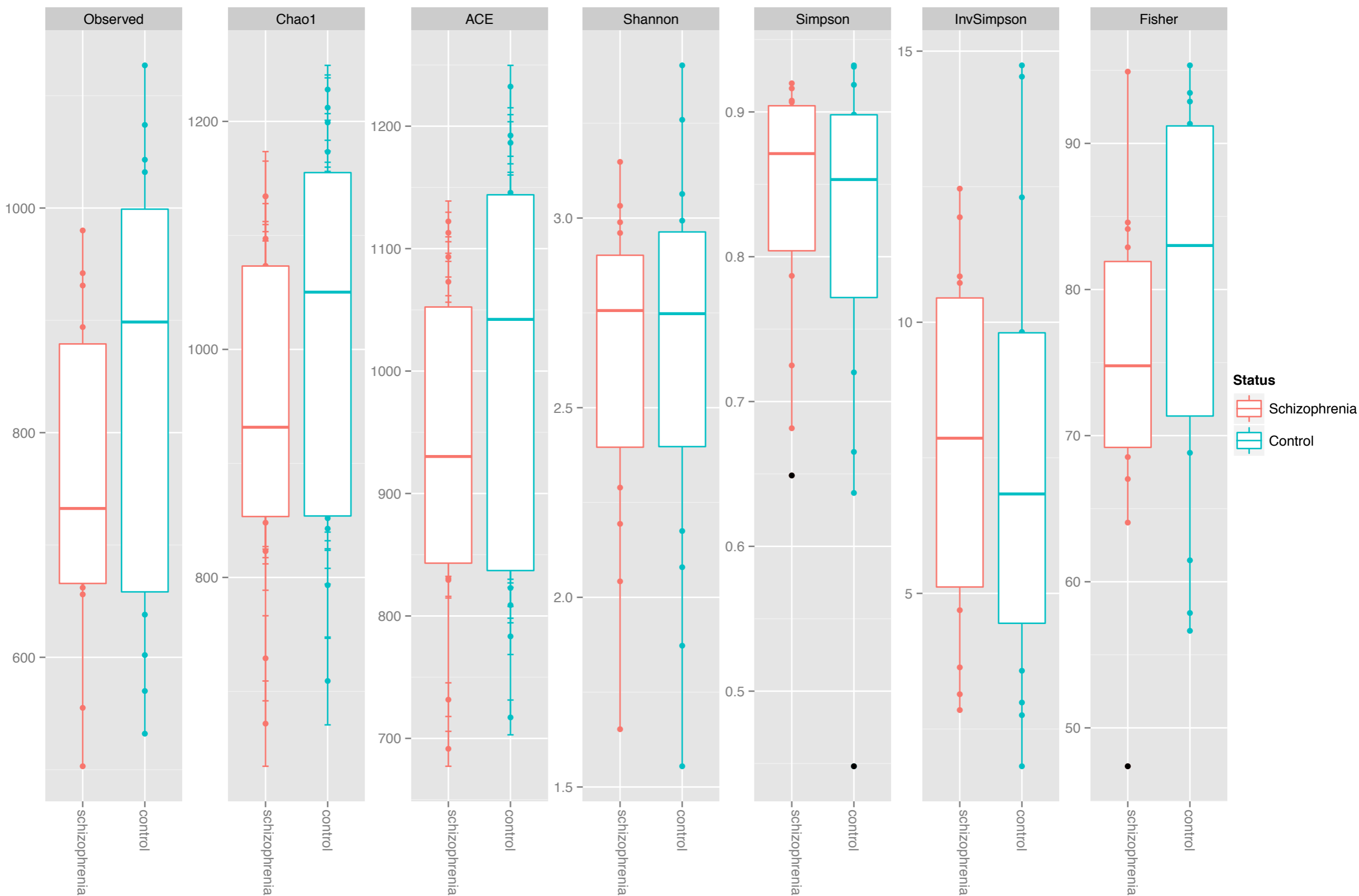**B****Phylum**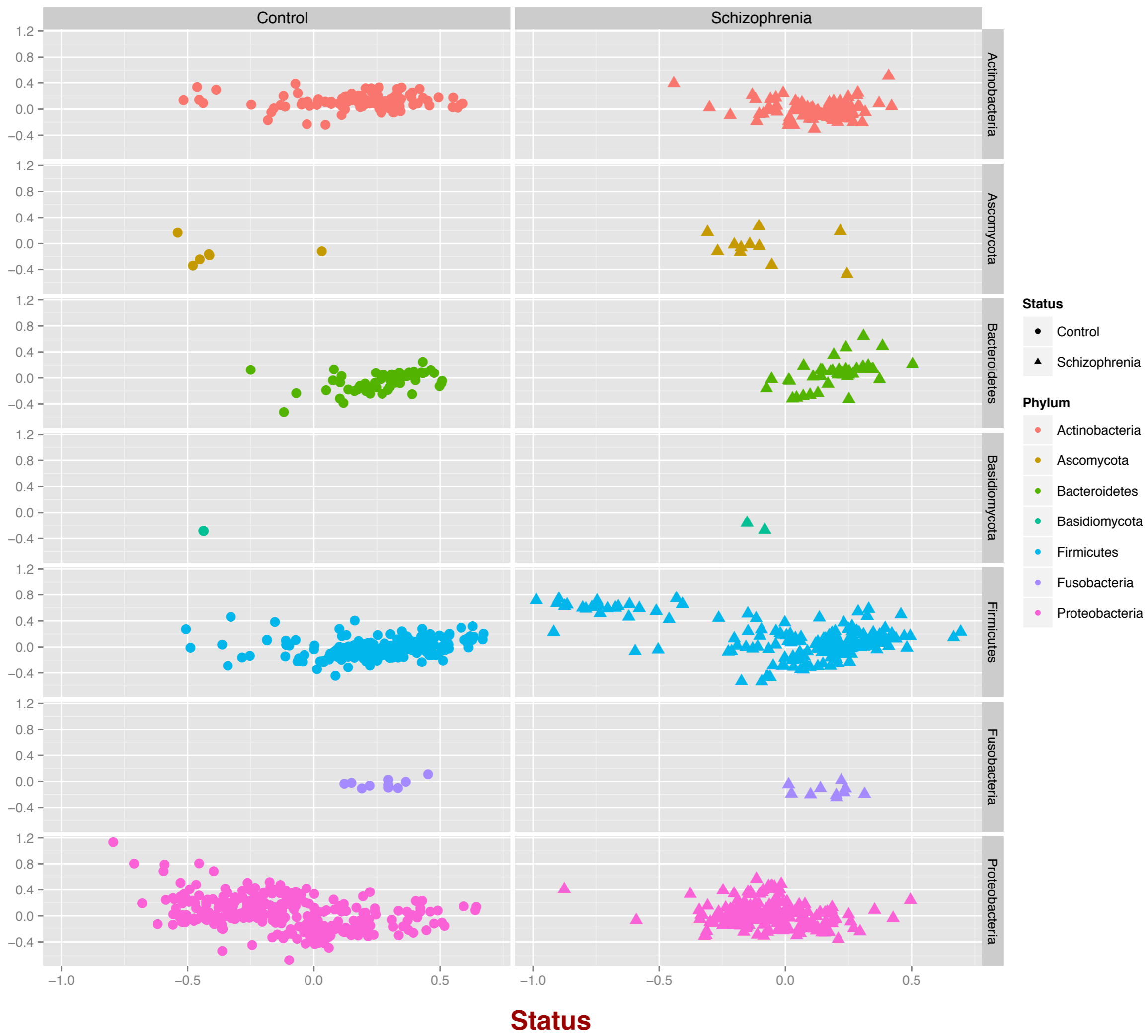

Supplement: Figure S1 — Schizophrenia samples are less rich in species than controls (Observed, Chao1, ACE indices) but are more homogeneously distributed (Shannon, Simpson, Inverse Simpson indices) as shown in A. In B, non-metric multidimensional scaling (NMDS; Bray-Curtis) shows that schizophrenia and control samples are similar in content at the phylum level; however, schizophrenia samples are more abundant in Ascomycota. [file peerj-03-1140-s001.pdf]

Effect Size (Log2 Fold Change)

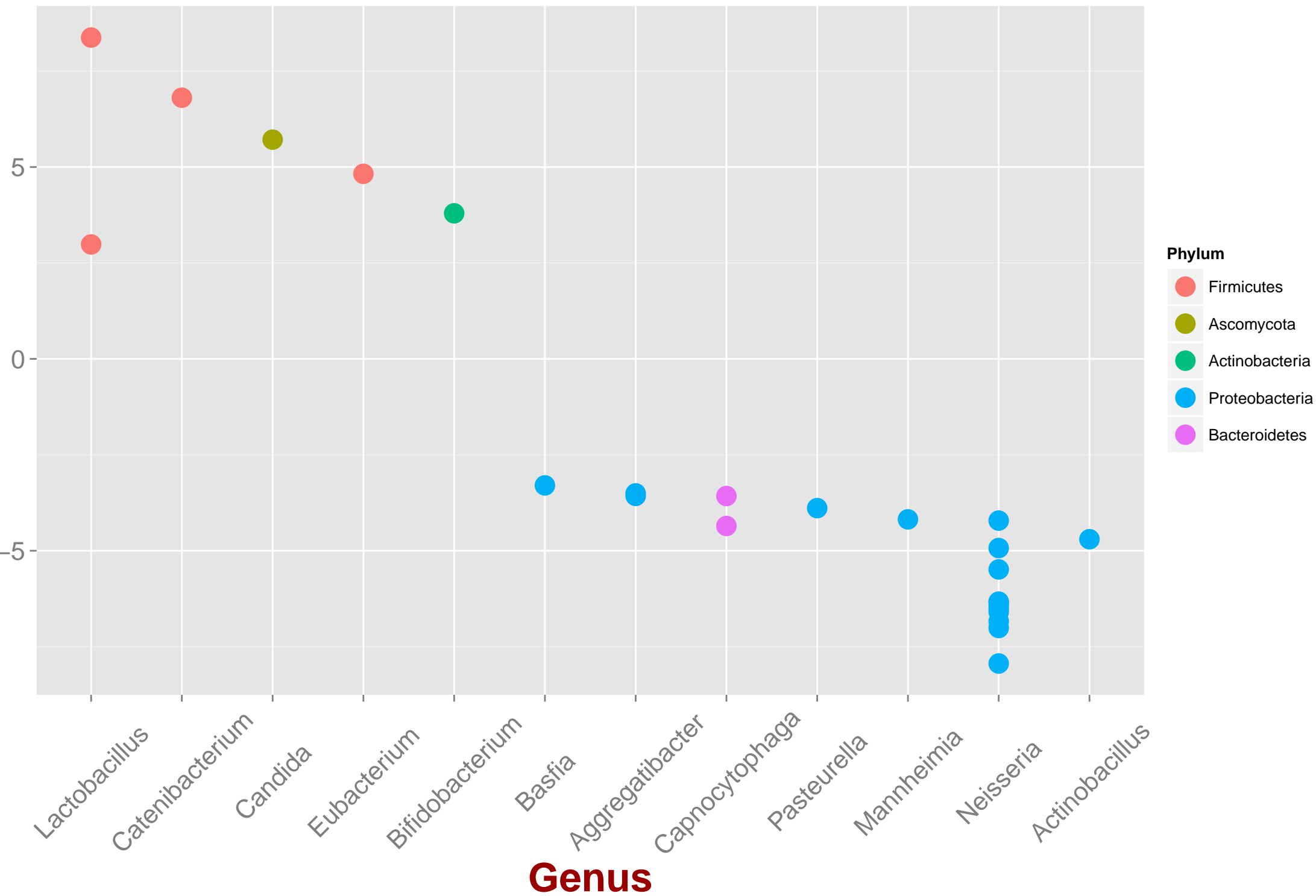

Supplement: Figure S2 — Species that are differentially abundant in schizophrenia and controls as shown by genus and color-coded by phylum. Dots represent species and y-axis is in Log2 scale. [file peerj-03-1140-s002.pdf]
